# Experiences of breastfeeding peer counseling among women with low incomes in the US: a qualitative evaluation

**DOI:** 10.1186/s12884-023-06213-0

**Published:** 2024-02-09

**Authors:** Elizabeth C. Rhodes, Mahrukh Zahid, Nafeesa Abuwala, Grace Damio, Helen Wilde LaPlant, Carrianne Crummett, Rebecca Surprenant, Rafael Pérez-Escamilla

**Affiliations:** 1 Hubert Department of Global Health, Rollins School of Public Health, Emory University, 1518 Clifton Rd, Atlanta, GA 30322 USA; 2 Yale School of Public Health, 135 College Street, New Haven, CT 06510 USA; 3 Hispanic Health Council, 175 Main Street, Hartford, CT 06106 USA; 4 Saint Francis Hospital and Medical Center, Trinity Health Of New England, 114 Woodland Street, Hartford, CT 06105 USA

**Keywords:** Breastfeeding, Community health worker, Health equity, Quality of care, Person-centered, Care experience, Qualitative research, United States

## Abstract

**Background:**

Person-centered breastfeeding counseling is a key but often overlooked aspect of high-quality services. We explored women’s experiences of the Breastfeeding Heritage and Pride™ program, an evidence-based breastfeeding peer counseling program serving women with low incomes in the United States.

**Methods:**

This study was conducted through an equitable community-clinical-academic partnership and guided by the World Health Organization (WHO) quality of care framework for maternal and newborn health, which highlights three domains of positive experiences of care: effective communication; respect and dignity; and emotional support. In-depth interviews were conducted with a purposive sample of women participating in the Breastfeeding Heritage and Pride™ program. Women were asked to describe their experiences with the program including examples of when good quality counseling was or was not provided. Each interview was conducted in English or Spanish, audio-recorded, and transcribed verbatim. Data were analyzed using reflexive thematic analysis. Once themes were generated, they were organized according to the three care experience domains in the WHO quality of care framework.

**Results:**

Twenty-eight in-depth interviews were conducted with a racially/ethnically and socio-economically diverse sample of women. Three themes described effective communication practices of peer counselors: tailoring communication to meet women’s individual needs; offering comprehensive and honest information about infant feeding; and being timely, proactive, and responsive in all communications across the maternity care continuum. Two themes captured why women felt respected. First, peer counselors were respectful in their interactions with women; they were courteous, patient, and non-judgmental and respected women’s infant feeding decisions. Second, peer counselors showed genuine interest in the well-being of women and their families, beyond breastfeeding. The key theme related to emotional support explored ways in which peer counselors offered encouragement to women, namely by affirming women’s efforts to breastfeed and by providing reassurance that alleviated their worries about breastfeeding. These positive experiences of counseling were appreciated by women.

**Conclusions:**

Women described having and valuing positive experiences in their interactions with peer counselors. Efforts to expand access to high-quality, person-centered breastfeeding counseling should, as part of quality assurance, include women’s feedback on their experiences of these services.

## Background

In the United States (US), there are large inequities in breastfeeding [1, 2]. Among women who gave birth in 2020, 19.7% of women with lower incomes exclusively breastfed through 6 months compared with 28.8% of women with higher incomes [3]. Among a longitudinal cohort of women with low incomes, Black and Hispanic women were less likely to meet their breastfeeding goals compared with white women [4]. These inequities are concerning given the many well-documented health benefits of breastfeeding for both women and their infants [5, 6]. Although there are many reasons why most women do not breastfeed as long as they want, one important barrier is poor access to high-quality breastfeeding support, particularly for women with low incomes and women of color [1, 4, 7, 8].

Breastfeeding peer counseling emerged as a key strategy for improving breastfeeding outcomes in the US when it was recommended in *The Surgeon General’s “Blueprint for Action on Breastfeeding”* in 2001 [9]. Breastfeeding peer counselors are women from a local community who have successfully breastfed and received training in breastfeeding education and management [10, 11]. They support their peers in making informed infant feeding decisions and anticipating and overcoming breastfeeding challenges to achieve their individual breastfeeding goals [10, 11]. In 2011, the *US Surgeon General’s Call to Action to Support Breastfeeding* identified breastfeeding peer counseling as a “core service” for women with low incomes participating in the Special Supplemental Nutrition Program for Women, Infants, and Children (WIC) [12], which serves over half of all infants born in the US [13]. In response, the US Department of Agriculture implemented *Loving Support© Through Peer Counseling: A Journey Together* nationwide [14, 15]. Community-based organizations and health systems across the country have also implemented breastfeeding peer counseling services for women with low incomes [16, 17]. Building on the *Biden-Harris Administration’s Maternal Health Blueprint*, the *Biden-Harris Administration National Strategy on Hunger, Nutrition, and Health* released in 2022 called for the expansion of breastfeeding support and counseling [18, 19].

In randomized controlled trials in both high-income and low- and middle-income countries, breastfeeding peer counseling has been shown to increase rates of breastfeeding initiation, duration, and exclusivity including among women living under conditions of poverty [10, 14, 15, 20–24]. Evidence from randomized controlled trials in the US demonstrate that breastfeeding peer counseling improves breastfeeding outcomes for women with low incomes, white women and women of color, and English and Spanish-speaking women [15, 20, 21, 25]. As a result of the consistent evidence available across world regions, the World Health Organization (WHO) has issued a guideline on breastfeeding counseling [11] and corresponding implementation guidance [26] that fully endorse breastfeeding peer counseling globally including in the US as an evidence-based intervention. Furthermore, the WHO recommends that breastfeeding peer counseling be person-centered [11], defined as care that is “respectful of and responsive to individual preferences, needs, and values” [27]. Both the WHO and the National Academies of Sciences, Engineering, and Medicine recognize person-centeredness as one of the six dimensions of quality of care [28]. However, the extent to which breastfeeding peer counseling programs in the US are person-centered is underexplored. Women’s interactions with health services, referred to as ‘experience of care,’ are an important part of high-quality, person-centered services [27]. While previous studies and reviews have explored women’s views and experiences with breastfeeding support [29–37], to our knowledge, there are few studies that focus on breastfeeding peer counseling, and those studies were not conducted in the US, were limited to the perspectives of peer counselors and staff, do not have the specific goal of evaluating the person-centeredness of these services, or use quantitative methods that do not generate an in-depth understanding of women’s counseling experiences.

To guide efforts to improve the quality of maternal and newborn health services within health systems including monitoring and evaluation of the person-centeredness of services, the WHO developed a conceptual framework of quality of care for pregnant women and newborns [38]. The WHO quality of care framework highlights experience of care as an important component of the process of care, along with provision of care. It identifies three key domains of quality to promote positive care experiences: effective communication with women and their families, care with respect and preservation of dignity, and emotional support. It also proposes that positive care experiences influence people-centered outcomes such as satisfaction. Guided by this framework, we conducted a qualitative study to describe women’s experiences of the Breastfeeding Heritage and Pride™ program (BHP), an evidence-based breastfeeding peer counseling program in Connecticut and Massachusetts serving predominantly women with low incomes many of whom self-identify as women of color [39]. Specifically, we explored women’s experiences across the three experience of care quality domains of the framework and organized the findings according to these domains. We expect that our findings will inform efforts to improve the person-centeredness of current and future breastfeeding counseling programs within health systems in similar contexts in the US. Furthermore, our methods including the application of the WHO quality of care framework to explore women’s experiences of breastfeeding peer counseling could be used in a variety of contexts both in the US and other countries.

## Methods

### Breastfeeding Heritage and Pride™ program

The process of co-designing BHP with community input, the person-centered program model, its implementation for over three decades, and its impact on breastfeeding outcomes have been described in detail elsewhere [39]. Briefly, the Hispanic Health Council, a community-based organization, partners with healthcare systems to deliver free breastfeeding counseling services in both clinical and community settings. Peer counselors are specialized community health workers who have successfully breastfed and completed training on key topics, such as: lactation management; the influence of social determinants of health on women’s abilities to meet their breastfeeding goals; and best practices for respectful communication and cross-cultural and diversity inclusiveness. Peer counselors recruit pregnant women from healthcare facilities through referrals from clinicians. Once enrolled, women receive breastfeeding education, anticipatory guidance, and lactation management support from peer counselors through individual in-person sessions supplemented by phone contacts. Counseling begins in pregnancy and continues up to 1 year postpartum. Program International Board Certified Lactation Consultants (IBCLCs) provide clinical guidance, as well as ongoing training and supportive supervision to peer counselors. Each year BHP serves over 1400 women in Connecticut and Massachusetts. Two randomized controlled trials demonstrated the efficacy and effectiveness of BHP in improving any and exclusive breastfeeding among predominantly women with low incomes who identify as Hispanic, Black, white, and other ethnicities [20, 21]. The US Centers for Disease Control and Prevention and National Academies of Sciences, Engineering, and Medicine endorsed BHP as an evidence-based program [40, 41]. In 2021, the WHO highlighted it as an exemplary program in its implementation guidance on breastfeeding counseling [26].

### Study team and reflexivity statement

This study was conducted through a partnership between Yale School of Public Health, the Hispanic Health Council, and Trinity Health Of New England. The team jointly developed the research objective, interview guide, participant recruitment protocol, and data collection and analysis plan. Researchers based at Yale with expertise in breastfeeding counseling and qualitative methods, along with two research assistants with master’s degrees in public health, recruited women participating in BHP and collected and analyzed the data. Researchers trained the research assistants in best practices for recruitment and qualitative data collection and analysis prior to the start of the study and provided supportive supervision to them throughout the research process [42]. Of the eight team members, four identify as people of color and three are bi-lingual in English and Spanish. All members of the research team were involved in or aware of previous research demonstrating the positive impact of BHP on breastfeeding outcomes. Members of the research team implementing BHP and researchers based at Yale believed BHP delivered high-quality counseling. The research assistants were not familiar with BHP prior to joining the research team.

Recognizing our collective knowledge and beliefs about the program, we were mindful to develop interview guide questions and probes that were neutral and would elicit information on both positive and negative counseling experiences. Researchers and research assistants at Yale who led the analysis and wrote the results practiced ongoing reflexivity by consciously and continuously reflecting on the ways in which our assumptions may shape the development of codes, coding of the data, and data interpretation [42]. To challenge our assumptions and maintain the validity of the findings, we stayed close to the data throughout the analytic process and writing of this manuscript.

### Study design, sampling, and recruitment

This study was part of an evaluation of the delivery of BHP during the COVID-19 pandemic [43]. We used a cross-sectional study design, which was appropriate for understanding women’s views about the program. Women were purposively sampled from a BHP enrollment list using a maximum variation approach to achieve diversity based on the health facility where they receive maternity care, assigned peer counselor, and race/ethnicity. Research assistants contacted women by phone and text and invited them to participate in an interview if they met two inclusion criteria: were aged 18 or older and spoke English and/or Spanish. Of the 61 women who were invited to participate, 19 refused and 42 agreed. Of the 42 who initially agreed, 28 were ultimately available to participate in an interview.

### Data collection and analysis

Both data collection and analysis were guided by the WHO quality of care framework for maternal and newborn health [38]. A semi-structured interview guide was developed in English and translated into Spanish by a fluent Spanish speaker and then checked and finalized by a native Spanish speaker. The guide included nine open-ended questions and probes to elicit women’s perspectives on the delivery of breastfeeding peer counseling before and during the COVID-19 pandemic. To contextualize women’s experiences of BHP, opening questions and probes at the beginning of the guide elicited women’s reasons for joining BHP and successes and difficulties with breastfeeding. Key questions and probes elicited women’s experiences of BHP with regards to the three domains of care experiences within the WHO quality of care framework: communication with peer counselors; respectful treatment by peer counselors; and emotional support from peer counselors. For example, the guide included questions like “How do you communicate with your peer counselor?” and “Are you able to communicate well with your peer counselor? Why/why not?”. Women were also asked to share the kind of support they received from peer counselors, examples of when good quality counseling was or was not provided by peer counselors, what aspects they liked most about BHP, and suggestions to make the program better. The interview guide was pilot tested in English and Spanish with women participating in BHP (*n* = 2 in English, *n* = 2 in Spanish) to assess issues such as whether participants understood the questions immediately, the order of questions was logical for participants, and the research questions could be answered with the information collected [42]. The guide was then refined, with the same refinements made to both the Spanish and English versions of the guide. For example, several questions were rephrased to improve clarity and new probes were added to help elicit in-depth information.

In-depth interviews were conducted between June and August 2020 by Zoom Video or Phone. Participants chose whether the interview was in English or Spanish. Interviews lasted approximately 30 to 45 minutes and were audio-recorded. A professional company produced verbatim transcripts in English, which were checked for accuracy by the study team. Each transcript was read closely and used to create a summary of the interview, using a structured notes template. As part of practicing reflexivity, researchers and research assistants held debriefing sessions to identify and reflect on initial assumptions about the quality of counseling provided by peer counselors and to check these assumptions against counseling experiences described by women. These sessions were also used to identify issues raised by women during interviews and make inductive refinements to the interview guide, such as the addition of probes to explore issues in greater depth in subsequent interviews [42]. Additionally, transcripts and transcript summaries were reviewed and discussed during debriefing sessions to assess saturation [44]. Once saturation had been reached, data collection stopped. Participants were mailed a letter and $30 gift card to acknowledge our gratitude for their participation.

Our analysis followed the phases of reflexive thematic analysis described by Braun and Clarke [45, 46]. First, the analysis team read and re-read transcripts to become immersed in the data. Making memos on the data and developing transcript summaries further enabled the team to become intimately familiar with the data. Next, codes were developed using both deductive strategies (e.g., topics from the interview guide related to the three experience of care domains in the WHO quality of care framework) and inductive strategies. All codes were organized into a codebook with code definitions and examples of when to apply each code [42]. All transcripts were then coded by one research assistant using MAXQDA18 (VERBI GmbH, Berlin). To ensure accurate and consistent application of codes across the entire dataset, coded transcripts were reviewed by the qualitative research lead and discussions were held to identify and resolve any disagreements in how codes were applied. After coding, initial themes were generated by writing detailed descriptions of each code that included an analytic narrative and quotations to vividly illustrate issues raised and give voice to participants and by identifying potential themes through team discussions. Next, the candidate themes were checked against the coded data segments and thick descriptions and further developed. Themes were then refined, which involved developing a detailed analysis of each theme. An informative name was also created for each theme through team discussions and consensus. To write the results, an analytic narrative for each theme was refined and illustrative quotations were selected for inclusion in the manuscript. This analytic approach involved an iterative process in which the analysis team moved back and forth between the phases. Throughout the analysis, the team re-read the textual data to ensure the themes and narrative text explored and captured the topics, views, and context raised by participants.

Finally, themes were organized according to the three domains of experience of care in the WHO quality of care framework for maternal and newborn health (effective communication; respect and dignity; and emotional support) [38]. In cases where topics within a given theme related to one or more domains, we reached consensus as a team about where to include the topic, taking into consideration the context in which women described the topic. For example, women noted that their peer counselors did not raise their voices in the context of describing respectful interactions. As such, we included this particular aspect within a theme under the respect domain, though not shouting could also be considered an ineffective communication method [47].

## Results

We interviewed 28 women who were socio-economically diverse (Table 1). Women ranged in age from 18 to 44 and identified as Black, white, bi- or multi-racial, and Hispanic/Latina. Over half of women had more than a high school education. Others were a high school graduate or had earned a general education diploma, with one woman having some high school. Over half of women had one child and reported not having previous experience breastfeeding before enrolling in BHP.

**Table 1:** Characteristics of women interviewed (*n* = 28)

Characteristics	*n* (%)
Age, years	
18–21	3 (10.7)
22–34	21 (75.0)
35–44	4 (14.3)
Race	
Black	12 (42.9)
White	7 (25.0)
Bi- or Multi-racial	2 (7.1)
Other	7 (25.0)
Hispanic/Latina	
Yes	12 (42.9)
No	16 (57.1)
Marital Status	
Single	3 (10.7)
With a partner (not married)	10 (35.7)
Married	15 (53.6)
Living with spouse/partner	
Yes	24 (85.7)
No	4 (14.3)
Education	
Some high school	1 (3.6)
High school graduate/general education diploma	10 (35.7)
More than high school	17 (60.7)
Parity	
1	16 (57.1)
> 1	12 (42.9)
Past breastfeeding experience	
Yes	11 (39.3)
No	17 (60.7)

We identified six themes that captured women’s experiences of counseling. Using the WHO quality of care framework as an organizing framework, we first present the themes regarding women’s experiences communicating with peer counselors, followed by a presentation of themes that explored women’s perspectives on how peer counselors treated them with respect and provided emotional support (Table 2). Many women reported difficulties with breastfeeding, such as challenges with latching, insufficient milk supply, breastfeeding while recovering from Cesarean sections, mastitis, and pain while breastfeeding. In this context, women described their experiences receiving breastfeeding education and support from peer counselors, both before and during the COVID-19 pandemic.

**Table 2:** Themes according to the domains of experiences of breastfeeding peer counseling. Women participating in the Breastfeeding Heritage and Pride™ program (*n* = 28)

Domains	Themes	Examples of practices that promoted positive counseling experiences
Effective communication	Tailoring communication to meet women’s individual needs	• Listened to women’s questions and concerns and responded with information that met their specific needs • Inquired about and documented details about women in order to tailor breastfeeding education and support to address their specific challenges and situations • Initiated conversations to learn about women’s needs for items necessary for breastfeeding and caring for their infants like breast pumps and diapers and then provided these resources • Practiced clear communication – for example, by using everyday words and offering clear explanations about infant feeding practices • Promoted women’s understanding of information by repeating information, explaining the same information in a variety of ways, and sharing videos and photographs to illustrate concepts like how to position infants while breastfeeding and get a good latch
	Offering comprehensive and honest information about infant feeding	• Offered comprehensive information by providing supplementary written materials and videos on infant feeding, and by sharing not only “textbook” information on infant feeding, but also personal views and experiences with breastfeeding • Exhibited honesty when sharing information about infant feeding
	Being timely, proactive, and responsive in all communications across the maternity care continuum	• Provided anticipatory guidance through a series of contacts and at times when it was most relevant and needed, beginning in pregnancy and continuing through the postpartum period • Reached out to women periodically to inquire about their breastfeeding experiences, address concerns, and assist with any challenges • Talked with women when it was convenient for them • Responded promptly to women when they had infant feeding questions or reached out for lactation support
Respect and dignity	Respectful breastfeeding counseling	• Interacted with women in a “courteous,” “friendly,” and “warm” manner • Did not raise their voices, “get mad” or frustrated, or use rude language • Demonstrated patience during counseling visits • Had a “non-judgmental” attitude when asking and responding to questions • Did not pressure women to join or continue participating in the program • Gave women space to decide when to reach out for additional support • Respected women’s infant feeding decisions, including their choices to partially or fully formula feed • Did not pressure women to breastfeed, while remaining supportive of breastfeeding • Did not criticize or make “mom shaming” comments about women’s infant feeding and care practices
	Being “genuinely interested” in women and their families	• Showed interest not only in women’s infants, but also in them as individuals who matter – for example, by asking women about their lives and their well-being • Knew and valued women as people and remembered details about their lives • Inquired about women’s family members and showed interest in infants beyond whether they were breastfeeding • Were attentive and spent time when offering support, rather than rushing
Emotional support	Offering encouragement to women	• Encouraged women to achieve their breastfeeding goals through praise and words of encouragement • Offered reassurance to alleviate women’s worries about breastfeeding

### Effective communication

We identified three themes relevant to the domain of effective communication: (1) tailoring communication to meet women’s individual needs; (2) offering comprehensive and honest information about infant feeding; and (3) being timely, proactive, and responsive in all communications across the maternity care continuum. These themes reflect the range of effective communication strategies practiced by peer counselors. Indeed, all women reported that they had good communication with their peer counselors.

#### Tailoring communication to meet women’s individual needs

This theme captures the multiple ways that peer counselors tailored their communication to meet women’s individual needs, which could be grouped into the type of information provided and the clear way in which the information was communicated.

In terms of the type of information provided, women reported that peer counselors offered information that was relevant for their specific needs. Women described peer counselors as “good listeners,” as peer counselors listened carefully to women’s questions and concerns and then responded with information to address them. Some women noted that their peer counselors asked and wrote down details about them like their breastfeeding difficulties, who might be helping with their infants, and the type of support they had for breastfeeding from family and friends. Peer counselors then used this information to tailor discussions to address their specific challenges and situations. In one case, however, a woman mentioned that her peer counselor sometimes shared information that she was already familiar with, such as information on how to latch even though she was familiar with latching because she had breastfed previously and latched successfully with her most recent baby. A few women shared that peer counselors initiated conversations to understand their needs for breastfeeding resources like breast pumps and breast pads to alleviate pain, diapers, and other necessary items to care for their infants. Peer counselors then provided these resources, which was helpful when women could not afford these items and did not have other sources of support. Additionally, many women shared that their peer counselors talked with them about their specific challenges related to breastfeeding like problems with tongue-tie and adverse social determinants of health like economic struggles, and then helped address these challenges. Women reported that this individualized support was very “encouraging,” and because it was tailored to their needs, it did not feel “overwhelming.”

Tailoring communication included practicing clear communication. Women frequently reported that peer counselors used everyday words and offered clear explanations about infant feeding practices that women could easily understand. Some women described additional ways that peer counselors promoted their understanding of breastfeeding-related information, such as repeating information, explaining the same information in different ways, and sharing videos and photographs to help convey information that is hard to describe in words alone:

*Communication was good because whenever I had a question for her, she would know how to answer and explain it to me in a way I would understand it well. When I asked her a question, for example, what is the best position or how can I achieve a better grasp, she would even send me videos for me to have a good idea. Or she’d send me photographs. She explained things very clearly, I was always able to understand it*. (Hispanic woman, 28 years, with previous breastfeeding experience).

For several women, having a trusted peer counselor who shared information in a clear and understandable manner was helpful because they felt overwhelmed and confused by all the breastfeeding information shared by friends and family members and the vast amount of information available online, especially when it was inconsistent:

*Really having somebody who is a sounding board and is able to listen and have current knowledge and not feel like you’re Googling everything because basically if you Google anything you’re dying it feels like. And there’s so much information online, and it feels very confusing and conflicting. So having somebody that, you know, I’m able to just talk to one person about whatever’s going on. That to me right there was just like, the gold, you know? That I didn’t have to go and double-check everything.* (white woman, 34 years, no previous breastfeeding experience).

#### Offering comprehensive and honest information about infant feeding

Women perceived the infant feeding information they received from peer counselors to be comprehensive and honest. They felt the information was comprehensive because peer counselors not only shared information during counseling sessions, but even provided supplementary written materials and videos. Additionally, peer counselors shared their own perspectives and experiences with breastfeeding, including their infant feeding decisions and breastfeeding challenges. Women found it useful to learn about their peer counselors’ experiences, particularly in the context of identifying with and knowing their peer counselors:

*She has information to share about her own personal experience, which is helpful too. It’s not like she’s just, you know, like, reading out of a book or, you know, providing just, like, the textbook information. But there’s information that she can share about her own personal experience, which is super helpful.* (non-Hispanic Black woman, 35 years, with previous breastfeeding experience).

Several women pointed out that peer counselors were honest when sharing information about infant feeding and their own breastfeeding experiences. Specifically, peer counselors offered truthful answers to women’s breastfeeding questions and acknowledged the difficulties of breastfeeding. For example, one woman shared her perspective that “breastfeeding is very good… from the fact that it saves you money because you don’t have to buy formula to your baby will be much healthier, but all that requires sacrifice.” She recounted that her peer counselor was forthright that breastfeeding would not be easy, which helped her feel more prepared.

#### Being timely, proactive, and responsive in all communications across the maternity care continuum

This theme captures how peer counselors provided timely anticipatory guidance, practiced proactive communication, and were available and responsive throughout the maternity care continuum. Women reported that peer counselors communicated with them regularly throughout pregnancy, immediately after childbirth, and in the postpartum period. They described three key aspects of this regular communication. First, peer counselors anticipated their needs and potential breastfeeding challenges and provided information that answered their questions even before they asked them. Women expressed appreciation that their peer counselors tracked and were aware of their stages of pregnancy, infants’ developmental milestones, upcoming pediatrician appointments, and other key time points, and then called “right on time” to offer anticipatory guidance. Women found it beneficial that peer counselors offered this anticipatory guidance over a series of multiple contacts and at times when it was most relevant and needed, rather than providing all the information at one time:

*I think every mother, whether you’re a first, second, or a third-time [mother], finds it helpful to be reminded of…what baby’s development, especially in regards to breastfeeding, should look like and as far as, like, feedings and all of that stuff. So, just being able to get the education in real-time. So not just, you know, being provided information in the beginning of or before I had the baby about what breastfeeding should look like, but being able to have those phone calls in real-time and ‘she is here at this stage and this is what it should look like’ is helpful.* (non-Hispanic Black woman, 35 years, with previous breastfeeding experience).

Second, peer counselors took a proactive approach in their communication with women, even beyond reaching out at critical time points to offer anticipatory guidance. This typically involved peer counselors reaching out to women periodically to inquire about their breastfeeding experiences, address any concerns that may have arisen, and assist with challenges. When women encountered difficulties with breastfeeding that required ongoing help, peer counselors also proactively communicated with women until the challenges were resolved. One woman who was infected with SARS-CoV-2 recounted that her peer counselor was in close touch with her during and after the infection to ensure she had the support to continue breastfeeding. In reflecting upon the communication with her peer counselor during this time, she shared, “It was very good, because she called me, she was on top of it, …she called me every other day to check how I was doing. When she didn’t call, she texted.”

Third, peer counselors were available and responsive when women reached out to them. Peer counselors reminded women that they could reach out any time to talk. One woman reported, “…she [peer counselor] tells me I can call her or send her a message with any concern, anything I need, and she will get back to me.” Women felt that their peer counselors were “always there” and “available” to talk and “always had an answer.”

*I never really have no one to turn to or ask…for information or question, and I could rely on her [peer counselor]. I could take my phone up, text her and say, ‘Hey, you know, I don’t know…’ and she will give me step-by-step things to do or tell me what to watch, what to read.* (non-Hispanic Black woman, 28 years, with previous breastfeeding experience).

Women commonly reported that peer counselors were quick to return missed calls or messages and answer women’s questions. This responsive communication made women feel like their peer counselors were reliable and understanding of their needs. A couple women reported that peer counselors were also “very flexible” with their schedules and available to talk at times that worked well for women. For example, one woman shared:

*I was able to speak with [my peer counselor] when it was convenient for me instead of it seeming like, you know, I had to do it at this day at this time, and I have a busy life right now. So, …it was convenient for me.* (non-Hispanic Black woman, 31 years, breastfeeding experience).

However, a couple women suggested that BHP could be improved by expanding the hours peer counselors are available to talk, since women may have breastfeeding issues arise and need to talk outside of business hours.

### Respect and dignity

Women felt that peer counselors treated them with respect and dignity, including by (1) delivering respectful breastfeeding counseling and (2) showing genuine interest in the well-being of women and their families beyond breastfeeding. When explaining why they felt respected by peer counselors, they shared stories and examples of respectful interactions with peer counselors, often contrasting them with examples of disrespectful treatment. Furthermore, women expressed appreciation for being “treated right” by their peer counselors.

#### Respectful breastfeeding counseling

Women viewed breastfeeding counseling as respectful given peer counselors’ demeanors and attitudes and their respect for women’s infant feeding choices. They frequently described the demeanor and attitudes of peer counselors that made them feel that they were being treated with respect. They brought up that peer counselors were “courteous,” “nice,” “friendly,” and “warm” when providing counseling. They explained that peer counselors did not shout, “get mad” or frustrated, or use rude language:

*To me, a courteous person speaks correctly, is respectful, tells you things that you want to know. In other words, she answers your question without an attitude, without being rude, without raising her voice. I meant, to me, that is being polite. Being respectful. For instance, you may ask someone a simple question – you don’t know the answer, but for the other person, it may be something simple, and they answer with contempt…However, she’s not like that…she is well-mannered.* (Hispanic woman, 36 years, no previous breastfeeding experience).

Peer counselors were also viewed as patient during counseling sessions. For example, one woman was grateful that when her baby would fuss her peer counselor would wait on the call until her baby calmed down and then resume the conversation. Several women reported that peer counselors took time to listen to them and talk about breastfeeding, rather than rushing conversations. One woman shared, “she [my peer counselor] just has a very nice, calm voice. She gives me time to respond. Doesn’t rush me. I’d say lots of patience.”

Women also reported that peer counselors had “non-judgmental attitudes.” In particular, they shared that peer counselors were non-judgmental in both how they asked and responded to questions. Women felt that peer counselors did not “judge” them for asking questions, meaning that they did not make women feel like a question they asked about infant feeding was “silly,” “stupid,” or “unnecessary.” One woman shared:

*She’s not judgmental…She doesn’t ever make you feel like you’re not doing things right or that was something stupid that you did. She never made me feel like that. Especially being somebody who is inexperienced and doesn’t really know, you know? Is kind of learning as this process goes on because every baby is different. She’s never made me feel bad for not understand or not knowing or asking questions, or making me feel stupid for asking questions or anything like that.* (white woman, 34 years, no previous breastfeeding experience).

Women reported that peer counselors respected their decisions, which engendered a sense of self-determination and autonomy. Women did not feel pressured to join or continue participating in BHP because peer counselors did not “push” the program on them. Instead, peer counselors presented peer counseling as a “source of information” and support that was available, but use of these services was completely voluntary. One woman who decided to not continue with the program shared that her peer counselor responded to this news by saying that “she understood and respected [the] decision” because she needed to do what was “best” for herself. Women also liked and appreciated that peer counselors gave them the space to decide when to reach out for additional support, while reminding women that they were willing to do whatever they could to help and would visit their homes or answer phone calls as needed.

Women also felt that peer counselors respected their infant feeding decisions, even if they decided to not exclusively breastfeed or stop breastfeeding. Many women identified examples of disrespect that they did not experience during their conversations with peer counselors about infant feeding decisions, implying – and in one case explicitly noting – that they expected peer counselors to not respect their decisions. For example, peer counselors did not give orders or “push” breastfeeding, even though they were supportive of breastfeeding. Peer counselors also did not criticize or make “mom shaming” comments about women’s infant feeding practices like supplementing with formula or other infant caring practices. Consequently, peer counselors did not make women feel like their decisions were wrong or “stupid.” Instead, peer counselors respected women’s choices and preferences:

*You know, she [peer counselor] - she’s never really pushed anything. I know she’s very, obviously, pro-breastfeeding, but, from the start—we ended up having to supplement with formula when we were in the hospital, and, for the first two or so weeks until my milk really came in and we got into a good groove. She, you know, didn’t really bat an eye about it, whereas, you know, I just felt like someone in that role would have kind of an automatic judgment about it, but she was just kinda, like, “No, you have to feed your baby. Whatever works for you is what works.* (white woman, 32 years, no breastfeeding experience).

When peer counselors listened to women and expressed an understanding of the challenges of breastfeeding, it further signified to women that peer counselors respected their decisions about how to feed their infants.

#### Being “genuinely interested” in women and their families

Women felt peer counselors were invested in their overall well-being as individuals who matter and in the well-being of their families. One way that peer counselors demonstrated that they truly cared about women was by asking them about their lives and well-being, as well as inquiring about their infants and partners. They explained that counseling calls began with greetings and peer counselors asking them how they were doing. Some women expressed gratitude that peer counselors not only showed interest in their infants, but also in them as individuals:

*She [peer counselor] just talks to me about how things are going. She asks me, like, about how I feel and stuff like that. And I don’t get that a lot ‘cause it’s more like, “How’s the baby? How’s the baby? How’s the baby?” It’s more like she actually cares about how I feel too, ‘cause I’m goin’ though stuff too. So, it’s like, she’ll call me, and we’ll talk for, like, a hour. And it’s really great!* (non-Hispanic Black woman, 20 years, with previous breastfeeding experience).

Other times, women pointed out that peer counselors were interested in the health and development of their infants beyond whether they were breastfeeding:

*Every time that she texts or calls to reach out, she asks how we’re doing. “How’s the baby doing? How big is he?” You know, just kinda asking, like, milestones. “Is he sitting up? Is he crawling?” Like, she seems to just really want to know how things are going beyond just, “Are you still breastfeeding? Is it going well?” You know, she’s thinking more holistically at our life.* (white woman, 32 years, no breastfeeding experience).

When peer counselors remembered details about women’s lives, it signaled to them that peer counselors knew and valued them as people. For example, one woman noted that, “She’ll [peer counselor will] ask how my daughter’s doing, how my husband’s doing …She remembers their names…She is remembering things.” In addition, women felt peer counselors were interested in them because peer counselors were attentive when they needed support. For example, one woman who was in the hospital for 4 days for childbirth shared that:

*…she would check back in every morning to see how everything was going. If I had any other questions, she always let me know that she was…within the hospital. Just send her a message, she’ll come as soon as she can*. (white woman, 34 years, no breastfeeding experience).

Another woman contrasted the attentiveness of her peer counselor with nurses who seemed to be in a hurry due to their high patient load:

*I felt like the nurses were, like, in a rush. They would just like, I don’t wanna say slap my baby on there, but they just, like, handed me my baby and be like, “Here put it—put her on.” And they’d tried, but like I said, I felt like they were in a rush. When the [peer] counselor walked in, she was with me for like, I’m not even exaggerating, I wanna say 2 hours trying to help me so my baby could latch.* (Hispanic woman, 27 years, no breastfeeding experience).

### Emotional support

Several women emphasized their desire and need for emotional support while breastfeeding, with one woman stating that emotional support was what she needed most. Women valued and appreciated peer counselors’ constant “emotional support,” especially postpartum given the breastfeeding challenges they encountered. In describing the emotional support peer counselors provided, women focused on how peer counselors offered encouragement. Women’s receipt of encouragement from peer counselors occurred within the context of peer counselors showing “understanding” and empathy for the breastfeeding challenges they faced.

#### Offering encouragement to women

Women received a great deal of encouragement from peer counselors, namely affirmation of their efforts to breastfeed and reassurance that alleviated their concerns about breastfeeding. Women recalled many instances where their peer counselors offered praise and words of encouragement like “Don’t give up. You’re doing great. I’m so proud of you.” Encouragement was helpful both for women who encountered breastfeeding difficulties as well as those who did not, with one woman sharing that “It wasn’t like I need a lotta help [with breastfeeding]. [Encouragement] was what I needed to get.” Peer counselors’ encouragement was particularly helpful for women who had no or limited social support for breastfeeding – for example, women who recently immigrated to the US and women whose partners, family members, and friends did not provide support, or suggested women “just quit” breastfeeding and feed their infants formula. For these women, receiving encouragement from peer counselors was instrumental in their efforts to breastfeed.

While women were excited about having a new baby, they were apprehensive about breastfeeding. When women shared their concerns about breastfeeding with their peer counselors, peer counselors took time to alleviate their concerns, including worries about insufficient milk, problems with latching, and pain while breastfeeding. Peer counselors addressed their concerns and reduced their anxiety not only by providing lactation management support to resolve breastfeeding difficulties but also by listening to women’s concerns and reassuring and comforting them:

*Every time she calls me I feel good, because I worry most of all about milk production—it doesn’t—I feel I need more but they tell me everything is fine, that as long as my baby—as long as I have milk, everything will be fine. As he gets older, I’m going to produce more and more milk. The thing about me is that I thought that as soon as my baby was born, I would have lots of milk and I didn’t. But they do provide a lot of peace of mind whenever I speak to them.* (Hispanic woman, 25 years, no previous breastfeeding experience).

Beyond breastfeeding, pregnancy and the postpartum period were viewed as an emotional and difficult period, making it particularly valuable to have emotional support from peer counselors during this time:

*All the emotion that you’re going – when you go through when you’re pregnant, all that, it was a lot for me, and I really needed somebody to talk to about it, talk to about all the – all whatever I was goin’ through. So that was helpful for me.* (non-Hispanic Black woman, 27 years, no previous breastfeeding experience).

## Discussion

This study shows that BHP breastfeeding peer counselors can, from the perspectives of women, deliver person-centered breastfeeding education and support. Women reported that peer counselors communicated effectively using an array of strategies and treated them with respect by providing respectful counseling and showing interest in women and their families. They also shared that emotional support offered by peer counselors encouraged them to continue breastfeeding and allayed their worries about breastfeeding.

This study has strong programmatic and policy implications for implementation of best practices outlined in the guideline on breastfeeding counseling issued by WHO and corresponding implementation guidance [11, 26]. Previous studies have focused largely on the impact of breastfeeding counseling on breastfeeding outcomes such as breastfeeding initiation, duration, and exclusivity, without focusing on service processes like counseling experiences, which is an important aspect of care quality in the WHO quality of care framework and a key measure of person-centeredness [10, 17, 27]. Additionally, previous studies have described pregnant, birthing, and postpartum women’s desires with regards to high-quality maternity care including breastfeeding support, as well as the mismatch between their needs and preferences and the implementation of maternity and breastfeeding services [47–50]. A novel contribution of our study is that it identified specific practices peer counselors employed to promote positive counseling experiences, from the perspectives of women.

The finding that peer counselors offered tailored information and support is noteworthy given results from previous qualitative studies that recommended having healthcare providers offer individualized maternal and child health services, including breastfeeding support, to promote positive experiences [34–36, 48]. Indeed, women in our study described a range of experiences from having no breastfeeding difficulties to facing multiple challenges as well as a variety of informational and emotional needs. This finding underscores the need for peer counselors who can provide individualized education and support to address each woman’s specific needs and preferences. Furthermore, the importance of clear communication has been described in the broader health care literature on effective patient-provider communication, as well as in the maternity care and breastfeeding literature [28, 34, 47]. A recent qualitative evidence synthesis on factors that influence women’s engagement with breastfeeding support conducted by Bengough and colleagues found that women did not want technical and clinical language that was inaccessible; instead, women reported a need for language that they could easily understand [29]. Clear communication is particularly important for promoting informed infant feeding decision-making among women with low incomes, a population with a high prevalence of low or limited health literacy [51].

Our finding that women appreciated peer counselors because they shared complete, truthful information about infant feeding is consistent with previous studies. For example, a recent qualitative analysis of text message conversations between women and WIC breastfeeding peer counselors found that women wanted to know about the mechanics of breastfeeding and sought advice for breastfeeding difficulties like engorgement and plugged ducts [52]. In a metasynthesis of 31 qualitative studies on women’s views and experiences of breastfeeding support in the US and other countries, Schmied and colleagues found that women wanted and appreciated accurate, sufficiently detailed information about breastfeeding, such as the range of benefits of breastfeeding, so that they could be in control of the decision to breastfeed [34]. Schmied and colleagues reported that women wanted realistic information, including information about potential breastfeeding difficulties [34]. According to the findings of this metasynthesis, women also viewed information that was not realistic, even if well-intentioned, to not be supportive, especially when they faced challenges with breastfeeding [34]. Powell and colleagues conducted a qualitative study with mothers, including some enrolled in WIC, and found that they perceived clinicians as not being honest with regards to potential breastfeeding difficulties [36]. Additionally, Powell and colleagues found that women wanted providers to be honest about the challenges that may arise, rather than only sharing the benefits of breastfeeding [36]. Similarly, a key finding from Bengough and colleagues’ qualitative evidence synthesis was that women wanted realistic information on both the benefits as well as the challenges of breastfeeding [29]. Messages like breastfeeding is easy and other idealistic information was perceived to hinder them from being prepared for breastfeeding issues that may arise like latching difficulties or mastitis [29].

Women in our study reported that peer counselors communicated in a timely and proactive manner, which was a positive but not surprising finding. BHP was designed to provide ongoing, well-timed breastfeeding education and support [39]. Peer counselors’ capacity to be available and responsive to women’s questions and need for support was likely facilitated by the Community Health Worker Caseload Estimator, a tool developed by the Hispanic Health Council to estimate the number of women each peer counselor can serve annually [39]. Peer counselors being available and responsive contrasts with previous literature that has documented women’s experiences interacting with hospital staff who were not available when women requested breastfeeding support [29].

In describing how peer counselors treated them with respect, many women offered examples of their previous disrespectful interactions with healthcare providers that they did not encounter when interacting with peer counselors. Women reported that peer counselors did not pressure them to breastfeed or criticize or judge them for supplementing with or switching to formula. Peer counselors’ ability to balance breastfeeding education and support with respect and acceptance of women’s infant feeding choices, as well as to empathize and reassure when breastfeeding did not go as hoped, is important. In their qualitative evidence synthesis, Bengough and colleagues highlighted that women perceived infant feeding messages conveyed by hospital staff to be biased towards breastfeeding and felt pressure to breastfeed [29]. Previous studies have found that women who formula feed can experience guilt, shame, and distress about their perceived failure to breastfeed, particularly in the context of breastfeeding promotion messages like “breast is best” and societal discourse that equates breastfeeding with being a “good mother” [53–55]. In a qualitative study of African American mothers and their support persons, Asiodu and colleagues reported that women teared up, softened their voices, and avoided eye contact when describing barriers to breastfeeding and described feelings of inadequacy, guilt, and shame for not being able to meet their prenatal breastfeeding goals [56]. Perceptions that healthcare providers, including peer supporters, promote breastfeeding as a moral obligation given its benefits for infant health, as well as negative responses to formula feeding from pro-breastfeeding providers, can leave women feeling inadequate and contribute to their feelings of guilt, shame, and regret when breastfeeding did not go as planned or recommended [53, 57–59]. The finding that BHP peer counselors created a supportive environment affirms that peer counselors successfully put into practice their BHP training on how social determinants of health influence infant feeding practices and the importance of showing empathy rather than contributing to “mother blame” narratives [60].

Women in this study highlighted that their peer counselors demonstrated patience during visits, consistent with previous qualitative studies in which women pointed out that an advantage of peer breastfeeding supporters was that they spend sufficient time with women to provide information and support focused on the needs of individual women and their babies [34]. This is noteworthy given evidence showing that women perceive healthcare providers like doctors and nurses to be rushed, which can make women feel guilty requesting help with breastfeeding or view healthcare providers as being unable or limited in their ability to help because they are preoccupied with other responsibilities [29, 34, 61].

Our findings strongly highlight the importance of providing women with emotional support, in addition to lactation management support. Women viewed the emotional support from peer counselors as critical, particularly during the postpartum period, which is consistent with studies examining experiences of doula services [62, 63]. Furthermore, findings from qualitative studies have shown that emotional breastfeeding support from healthcare providers is valued by women and influences the extent to which women feel supported [61]. Moreover, emotional support was meaningful for women who did and did not face breastfeeding difficulties. Thus, our study adds to the evidence indicating that emotional support should be an integral aspect of breastfeeding care through peer counseling and other approaches.

### Strengths and limitations

This qualitative study has several strengths. Since this study included a diverse sample of women with low incomes belonging to ethnic/racial groups that have historically been discriminated against, the themes that were generated may have strong transferability to women of color with low incomes in other regions of the US, though future research is needed to confirm this. To enhance the credibility of the research, reflexivity was practiced by the study team throughout the research process from the study design to data collection, interpretation, and presentation [42]. Additionally, this research was conducted as part of an equitable academic-community-clinical partnership guided by the principles of respectful community-based participatory research [64, 65].

This study also has some limitations. Because data were collected retrospectively, it is possible that women did not fully recall their interactions with their peer counselors. However, women were currently participating in BHP at the time of the interview and therefore would have had recent encounters with peer counselors. Data were collected during the COVID-19 pandemic when BHP was delivering services virtually via video and phone calls [43]. As such, the findings may not fully reflect women’s experiences of the program in non-emergency contexts. Of note is that many women not only shared information about their experiences interacting with peer counselors during the pandemic but also shared their experiences before the pandemic when peer counselors provided counseling in women’s homes and in healthcare facilities. We did not recruit women who dropped out of the program. Consequently, we may have missed experiences of women who stopped receiving services because they had a negative experience in their interactions with peer counselors or were dissatisfied with the program.

### Implications for breastfeeding counseling

Our overall finding that women had positive experiences of BHP underscores the value of co-designing breastfeeding peer counseling with communities and intentionally co-implementing them to meet the needs, values, and preferences of women and their families. It also highlights the importance of comprehensive training and supportive supervision of peer counselors not only to build their capacity to share breastfeeding information and provide lactation management, but also in understanding the social determinants of health that influence breastfeeding practices and in practicing respectful communication. The BHP protocol, which allows for meeting women where it works best for them and providing services according to their needs and as intensively as needed, can serve as a model for promoting person-centeredness in breastfeeding education and support within health systems [39].

Since person-centeredness is a key dimension of high-quality care, experiences of breastfeeding peer counseling across the different domains explored in this study should be measured for a complete understanding of service quality [28, 38]. To promote high-quality health systems, program monitoring and evaluation systems should track women’s experiences of breastfeeding counseling over time and use the data to hold implementers accountable to the women and families they serve as well as to target quality improvement efforts [27].

The reliance of breastfeeding counseling programs on private funding poses a major challenge to widespread program dissemination and sustainability. Based on evidence that breastfeeding counseling promotes equity in breastfeeding, the Hispanic Health Council played an active role in advising Connecticut’s efforts to secure Medicaid coverage for breastfeeding counseling services. Our findings contribute to the case for reimbursement. The results suggest that breastfeeding counseling is a solution for increasing the person-centeredness of breastfeeding education and support within health systems. Interventions to improve this aspect of breastfeeding service quality are of critical importance because all women have the right to be treated with respect and dignity and receive person-centered breastfeeding services [66, 67]. Moreover, mistreatment during interactions with healthcare providers in the course of breastfeeding care in health facilities, including experiences of racism and discrimination, is gaining attention as a driver of breastfeeding inequities. Several recent studies found that both women of color and healthcare providers reported racial/ethnic discrimination in breastfeeding care [68–71]. Examples of discrimination included healthcare providers assuming women of color will not breastfeed, providing less breastfeeding information and support to women of color compared with their white counterparts, ignoring women’s requests for help with breastfeeding difficulties, and racist remarks [68–71]. Emerging evidence also suggests that poor breastfeeding care experiences such as discrimination may negatively affect both breastfeeding initiation and duration [68, 71]. Therefore, systems-level solutions such as the integration and implementation of breastfeeding peer counseling within health systems that promote positive experiences, especially for racially and socio-economically marginalized populations, are pressing [1].

### Research recommendations

As efforts to increase the quality of breastfeeding counseling progress, it is critical to incorporate a focus on delivering services that are person-centered [72]. To advance this agenda, evaluations of WIC’s *Loving Support* program and other breastfeeding peer counseling programs are needed to deepen our understanding of women’s experiences of these programs [73]. These evaluations could also identify common features of person-centered programs across different contexts and populations. This recommendation has been in place for many years, but studies still need to be conducted to carry it out [73]. Future investigations are also needed to elucidate the influence of women’s experiences on their satisfaction with breastfeeding counseling services and their ability to meet their breastfeeding goals. Lastly, our study provides evidence that BHP offers person-centered breastfeeding counseling. However, the extent to which integrating peer counselors into the health system influences women’s overall experiences and satisfaction with facility-based breastfeeding care is unknown and warrants future research. Such research is of high relevance to health systems, since performance-based financial incentives are increasingly linked to patient experience scores [74, 75].

## Conclusions

Women in this study reported positive experiences of BHP, which provides training and supportive supervision of peer counselors combined with a flexible service protocol that enables peer counselors to deliver person-centered services [39]. This study calls for further implementation and systems research on high-quality, person-centered breastfeeding peer counseling. Policy makers and program implementers should prioritize women’s voices in breastfeeding peer counseling program co-design, implementation, and scale-up, and in quality improvement efforts within health systems.

## Data Availability

The qualitative dataset generated and analyzed during the current study are not publicly available, since sharing the data publicly could compromise the anonymity of participants. For questions about accessing the data, please contact the corresponding author.

## Abbreviations

BHP: Breastfeeding Heritage and Pride™ program
WIC: Special Supplemental Nutrition Program for Women, Infants, and Children
US: United States
